# Overexpression of the *VvSWEET4* Transporter in Grapevine Hairy Roots Increases Sugar Transport and Contents and Enhances Resistance to *Pythium irregulare*, a Soilborne Pathogen

**DOI:** 10.3389/fpls.2019.00884

**Published:** 2019-07-09

**Authors:** Eloïse Meteier, Sylvain La Camera, Mary-Lorène Goddard, Hélène Laloue, Pere Mestre, Julie Chong

**Affiliations:** ^1^Laboratoire Vigne, Biotechnologies et Environnement (LVBE, EA3991), Université de Haute-Alsace, Colmar, France; ^2^UMR CNRS 7267, Laboratoire Ecologie et Biologie des Interactions, Equipe “SEVE-Sucres et Echanges Végétaux-Environnement,” Université de Poitiers, Poitiers, France; ^3^CNRS, LIMA, UMR 7042, Laboratoire d’Innovation Moléculaire et Applications, Université de Haute-Alsace, Université de Strasbourg, Mulhouse, France; ^4^SVQV, Université de Strasbourg, INRA, Colmar, France

**Keywords:** grapevine, SWEET transporter, sugar, pathogen interaction, flavonoid

## Abstract

Sugar transport and partitioning play key roles in the regulation of plant development and responses to biotic and abiotic factors. During plant/pathogen interactions, there is a competition for sugar that is controlled by membrane transporters and their regulation is decisive for the outcome of the interaction. SWEET sugar transporters are the targets of extracellular pathogens, which modify their expression to acquire the sugars necessary to their growth (Chen et al., 2010). The regulation of carbon allocation and sugar partitioning in the interaction between grapevine (*Vitis vinifera*) and its pathogens is poorly understood. We previously characterized the SWEET family in *V. vinifera* and showed that SWEET4 could be involved in resistance to the necrotrophic fungus *Botrytis cinerea* in Arabidopsis (Chong et al., 2014). To study the role of VvSWEET4 in grapevine, we produced *V. vinifera* cv. Syrah hairy roots overexpressing *VvSWEET4* under the control of the CaMV 35S promoter (*VvSWEET4*_OX_). High levels of *VvSWEET4* expression in hairy roots resulted in enhanced growth on media containing glucose or sucrose and increased contents in glucose and fructose. Sugar uptake assays further showed an improved glucose absorption in *VvSWEET4* overexpressors. In parallel, we observed that *VvSWEET4* expression was significantly induced after infection of wild type grapevine hairy roots with *Pythium irregulare*, a soilborne necrotrophic pathogen. Importantly, grapevine hairy roots overexpressing *VvSWEET4* exhibited an improved resistance level to *P. irregulare* infection. This resistance phenotype was associated with higher glucose pools in roots after infection, higher constitutive expression of several genes involved in flavonoid biosynthesis, and higher flavanol contents. We propose that high sugar levels in *VvSWEET4*_OX_ hairy roots provides a better support to the increased energy demand during pathogen infection. In addition, high sugar levels promote biosynthesis of flavonoids with antifungal properties. Overall, this work highlights the key role of sugar transport mediated by SWEET transporters for secondary metabolism regulation and pathogen resistance in grapevine.

## Introduction

Grapevine (*Vitis vinifera*) is an economically important crop that is susceptible to diverse pathogens. Current strategies to control diseases in grapevine rely on the massive use of pesticides, making viticulture a major consumer of chemicals in agriculture, with important economic and environmental consequences. Source to sink transport and allocation of sugars are major determinants in the control of crop productivity and play key roles in the regulation of plant responses to biotic and abiotic factors (Lemoine et al., 2013). Since biotrophic and necrotrophic pathogens act as additional sinks within the host tissue, sugar transport and partitioning are modified following infection (Lemoine et al., 2013). Increasing evidence shows that during plant-pathogen coevolution, microorganisms have evolved sophisticated mechanisms to highjack sugar fluxes from their hosts (Baker et al., 2012). In grapevine, regulation of sugar allocation during the interaction with pathogens is poorly understood.

In plants, apoplastic sugar levels are controlled by glycoside hydrolases and membrane transporters regulating the levels of nutrients provided to the pathogen (Bezrutczyk et al., 2018). Enhanced sugar efflux from host cells and invertase activity can lead to sucrose and hexose accumulation into the apoplast, which are taken up by fungal sugar transporters (Lemoine et al., 2013; Veillet et al., 2016). Increased invertase activity and plant monosaccharide transporter expression have indeed been reported in several plant-pathogen interactions (Fotopoulos et al., 2003; Doidy et al., 2012). In *Arabidopsis thaliana*, apoplastic hexose retrieval mediated by the activity of Sugar Transport Protein (STP) is an important component of disease resistance. Whereas AtSTP13-deficient plants exhibit a reduced rate of glucose uptake and an enhanced susceptibility to *B. cinerea*, plants with a high constitutive level of AtSTP13 protein have improved capacity to absorb glucose and a better resistance to the fungus (Lemonnier et al., 2014).

On another side, it has also been proposed that sugars could represent signals for the induction of defense genes (Herbers et al., 1996; Herbers and Sonnewald, 1998; Gebauer et al., 2017). Several studies reported that high sugar levels in plant tissues are associated with a high level of resistance to fungal infection (Morkunas and Ratajczak, 2014). It has been hypothesized that sugars could provide the energy source to fuel defense responses and could also interact with hormonal signaling pathways regulating immune responses. Indeed, addition of sugars to plant culture media induced oxidative burst, lignification of cell walls, synthesis of flavonoids and PR protein expression (Morkunas and Ratajczak, 2014).

A class of sugar transporters playing a crucial role in plant-pathogen interactions is the SWEET family of sugar transporters. This family of sugar facilitators (PFAM code PF03083) comprises 17 members in Arabidopsis and 21 in rice (Chen et al., 2010). SWEETs are integral membrane proteins, with seven transmembrane domains, involved in sugar diffusion across membranes and playing important roles in nectar production as well as in seed and pollen development. Remarkably, SWEET transporters are targeted by extracellular pathogens, which modify their expression to obtain the sugar necessary for their growth (Chen et al., 2010). The role of SWEET proteins in plant resistance to pathogens has been well studied in rice. In this crop plant, the pathogenic bacteria *Xanthomonas oryzae* pv. *oryzae* uses different TAL (Transcription Activator-Like) effectors to up-regulate the expression of *SWEET* genes, resulting in sugar efflux into the apoplast, which supports the pathogen growth. The rice protein Xa13 is a SWEET transporter essential for pollen development and is also required for *X. oryzae* infection, Xa13-deficient plants being resistant to this bacterial pathogen (Chu et al., 2006). Several *xa13* alleles conferring recessive resistance to *X. oryzae* have been identified. In all cases resistance is associated with a mutation in the *Xa13* promoter (Chu et al., 2006; Römer et al., 2010). In rice, three out of 21 *SWEET* genes are targeted by pathogenic *X. oryzae* pv. *oryzae* (Yuan et al., 2014). This supports the general hypothesis that *X. oryzae* pv. *oryzae* induces developmentally regulated host genes to cause disease susceptibility. A similar mechanism has been described for the citrus bacterial canker disease caused by *Xanthomonas citri* subspecies *citri* (Hu et al., 2014) and the cotton blight caused by *X. citri* subsp. *malvacearum* (Cox et al., 2017).

Whereas the role of plant SWEET transporters is well characterized in the interaction with pathogenic bacteria, it is less documented in other pathosystems. In Arabidopsis, *SWEETs* are differentially regulated following infection with different types of pathogens (Chen et al., 2010), suggesting that pathogens with different lifestyles deploy specific strategies to divert host carbohydrates for their growth (Slewinski, 2011). AtSWEET2, which localizes to the root tonoplast, is involved in glucose sequestration in root vacuoles and limits carbon efflux from roots. Loss of function in *AtSWEET2* leads to enhanced susceptibility to the common root pathogen *Pythium irregulare*. This transporter has thus an important role in sugar retention and carbon availability modulation, with important consequences for root pathogen resistance (Chen et al., 2015).

Sugar partitioning is crucial in the major fruit crop grapevine, since berries accumulate high levels of sugars, which constitute a major component of fruit quality. Despite the importance of sugar allocation in grape berries, regulation of carbon allocation and sugar partitioning in the interaction with different pathogens is poorly understood. Hayes et al. (2010) reported an enhanced expression of the hexose transporter *VvHT5* and of a cell wall invertase, both regulated by abscisic acid, during the infection with biotrophic pathogens. Similarly, Gamm et al. (2011) showed an increase in both invertase expression and soluble sugar contents in leaves infected with the biotrophic oomycete *Plasmopara viticola*.

Previous work from our laboratory identified 17 *SWEET* genes in the *V. vinifera* 40024 genome and showed that they are differentially expressed in different vegetative and reproductive organs, as well as after grapevine infection with biotrophic (*Erysiphe necator*, *P. viticola*) and necrotrophic (*B. cinerea*) pathogens (Chong et al., 2014). Our results showed a strong up-regulation of *VvSWEET4* upon infection with *B. cinerea*. Moreover, *A. thaliana* knockout mutants in the orthologous *AtSWEET4* were found more resistant to *B. cinerea*, suggesting a role of VvSWEET4 in resistance to necrotrophs in grapevine (Chong et al., 2014). In this study, we investigated the role of VvSWEET4 in a homologous system by stable overexpression in hairy roots of *V. vinifera* cv. Syrah. We show that high levels of *VvSWEET4* expression resulted in enhanced root growth, higher glucose and fructose contents and higher radiolabeled glucose passive uptake. We further demonstrate that hairy roots overexpressing *VvSWEET4* are more resistant to *P. irregulare*, a common root pathogen. Enhanced resistance could result from high sugar levels and/or constitutive enhanced flavonoid biosynthesis.

## Materials and Methods

### Biological Material

*Agrobacterium rhizogenes* A4 strain (Tepfer, 1984) was maintained on MG/L medium as described in Torregrosa et al. (2015).

*Vitis vinifera* cv. Syrah *in vitro* plantlets were cultured on Mc Cown woody plant medium including vitamins (Duchefa, pH 6.2) supplemented with 15 g. L^–1^ sucrose and 0.7% bacto-agar in a growth chamber at 25°C, under a 16/8 h photoperiod and were subcultured every 2 months.

*Vitis vinifera* cv. Syrah hairy roots were cultured in Petri dishes on LG0 medium (Torregrosa and Bouquet, 1997; Torregrosa et al., 2015) in a growth chamber at 25°C, under a 16/8 h photoperiod. Every 3 weeks, root tip fragments of 1–2 cm length were harvested and subcultured on fresh LG0 medium.

*Pythium irregulare* strain isolated from carrot was provided by the Phytodiagnostique research and development centre of Vegepolys (Angers, France) and subcultured every week on PDA medium.

### Transformation of *V. vinifera* cv. Syrah With *Agrobacterium rhizogenes* and Hairy Roots Regeneration

*Agrobacterium rhizogenes* A4 strain (Tepfer, 1984) was transformed by electroporation with empty pBin61 plasmid or pBin61 containing *VvSWEET4* cDNA under the control of the 35S cauliflower mosaic virus promoter obtained as described in Chong et al. (2014). Four to six-week-old plantlets of *V. vinifera* cv. Syrah were used for *A. rhizogenes* transformation. Hairy root lines were obtained after transformation of stems of *in vitro* plantlets with *A. rhizogenes* as described in Torregrosa et al. (2015). Different hairy root lines were regenerated: lines transformed with pBin61 containing *35S::VvSWEET4* (*VvSWEET4*_OX_), lines transformed with empty pBin61 (pBin) and lines only transformed with *A. rhizogenes* A4 strain (A4).

### Measurement of Hairy Root Growth

Four to six root tip fragments of approximately 1 cm length were harvested from 3-week-old hairy roots and placed at the center of 9 cm diameter Petri dishes. The initial position of the root tip was marked on the plate. Hairy roots were cultured at 25°C under a 16/8 h photoperiod. Pictures of the roots were taken 4, 7, 11, and 14 days after subculture. Primary root length was measured from the marked start position with the Image J software as described in Corrales et al. (2014).

### Soluble Sugar Analysis

Frozen ground *V. vinifera* cv. Syrah hairy root tips (approximately 150 mg fresh weight) were extracted with 1.5 mL of methanol/chloroform/water (60/25/15, v/v/v). After vortex, the mixture was centrifuged at 5000 *g* for 10 min at 20°C. Supernatant was collected and pellet was reextracted 2 times with 900 μL of methanol/chloroform/water (60/25/15, v/v/v). Supernatants were pooled and mixed with 1.8 mL of water and centrifuged at 1200 *g* for 15 min at 20°C. The supernatant was collected and evaporated in a centrifugal vacuum evaporator (Eppendorf, 5301 concentrator) at 50°C for 3 h. The soluble glucose, fructose and sucrose contents of sample extracts were measured using the Sucrose/D-Fructose/D-Glucose Assay Kit (Megazyme) according to the manufacturer instructions.

### Measure of Invertase Activities

Cytoplasmic (CIN), vacuolar (VIN), and cell wall (CWIN) invertase activities were extracted and determined as described in Veillet et al. (2016).

### Sugar Uptake Assays With Radiolabeled Glucose

The sugar uptake assay was adapted from Veillet et al. (2016). Five primary root fragments of the same age (30-day-old) and size were placed for 60 min (2 times) in 6-well plate containing the equilibration buffer (20 mM MES-KOH pH 5.8, 1 mM CaCl_2_) under agitation. After equilibration, samples were transferred into the incubation buffer (20 mM MES pH 5.8, 1 mM CaCl_2_, 10 mM glucose) containing radiolabeled glucose (0.5 μCi. ml^–1^ of D-[U-^14^C]-glucose) for 15 min under agitation. To measure CCCP- independent glucose uptake (diffusion), CCCP (20 μM) was added into the equilibration buffer 10 min before addition of incubation buffer. After incubation, samples were washed three times for 2 min in equilibration buffer. Samples were left overnight in the digestion buffer (36.4% perchloric acid w/v, 0.017% triton X-100 w/v and 8.1% hydrogen peroxide w/v) at 60°C. Incorporated radioactivity was determined by liquid scintillation counting (Tri-Carb 2910 PR, PerkinElmer). Active glucose uptake results from the difference between total uptake and diffusion. Four independent repetitions have been realized with three technical replicates each comprising five roots for each repetition.

### Inoculation of Hairy Roots With *Pythium irregulare* and *in planta* Quantification of Pathogen Growth

Eight hairy root tip fragments of 2 cm length and approximately the same diameter were selected, cut and placed 2 cm apart in square Petri dishes containing water agar (8 g/L) medium to favor infection. Water agar plates contain low levels of free sugars (approximately 2 mg.L^–1^ sucrose, 0.16 mg.L^–1^ fructose, and 0.2 mg.L^–1^ glucose). One mycelium plug (0.5 cm^2^ square) from the edge of a 1-week-old *P. irregulare* culture was placed at equal distance between two root fragments. Square Petri dishes were partly covered with aluminum foil and incubated in upright position inside a growth chamber at 22°C with a 12 h photoperiod. The experiment was repeated 3 times.

Real-time quantitative PCR has been reported as an accurate method to monitor fungal development in plant tissues (Gachon and Saindrenan, 2004) and specific primers from the ITS region have been developed for *P. irregulare* detection (Spies et al., 2011b). *P. irregulare* growth in roots was determined by relative quantification of fungal and plant DNA by means of qPCR analysis. Total fungal and plant DNA were extracted from 8 root tips 3 days after pathogen inoculation as described (Gachon and Saindrenan, 2004). The relative quantity of *P. irregulare* was calculated according to the abundance of the oomycete *ITS* sequence (Spies et al., 2011b) relative to the grapevine-specific *ACTIN* and *EF1*α genes measured by qPCR as described in Berr et al. (2010). qPCR reactions were performed as described in the Section “Gene Expression Analysis by Real-Time Quantitative RT-PCR.” Primers used for real-time quantitative PCR are listed in Supplementary Table S1.

### Gene Expression Analysis by Real-Time Quantitative RT-PCR

RNA extraction and DNase I treatment were performed as described in Chong et al. (2008). Reverse transcription was performed on 1 μg RNA using the SuperScript II Reverse Transcriptase (Invitrogen) and oligodT priming as recommended by the supplier.

Real-time PCR reactions were carried out on the CFX96 system (Biorad, France). PCR reactions were carried out in duplicates in a reaction buffer containing 1X iQ SYBR^®^ Green Supermix, 0.2 mM of forward and reverse primers, and 10 ng of reverse transcribed RNA in a final volume of 25 μL. Thermal cycling conditions were: 30 s at 95°C followed by 40 cycles of 15 s at 94°C, 30 s at 60°C, and 30 s at 72°C. The calibration curve for each gene was obtained by performing real-time PCR with serial dilutions of the purified PCR product (from 10^2^ to 10^8^ cDNA copy number). The specificity of the individual PCR amplification was checked using a heat dissociation curve from 55 to 95°C following the final cycle of the PCR and by sequencing the final PCR products. The results obtained for each gene of interest were normalized to the expression of two reference genes (see legend of Figures) as described in Vandesompele et al. (2002) and relative expression (fold induction) compared to appropriate controls (see legend of Figures) was calculated as described by Pfaffl (2001). Mean values and standard deviations were obtained from two technical and three biological replicates. Primers used for real-time quantitative PCR are listed in Supplementary Table S1.

### Determination of Tannins With the Vanillin Method

Vanillin assay is quite specific to a narrow range of flavanols (monomers and polymers) and dihydrochalcones that have a single bond at the 2,3-position and free meta-oriented hydroxy groups on the B ring (Sun et al., 1998). Frozen ground *V. vinifera* cv. Syrah hairy root tips (approximately 150 mg fresh weight) were extracted twice with 80% methanol. After each extraction, the mixture was centrifuged at 10 000 *g* for 10 min at 20°C. Supernatants of both extractions were pooled. The content of tannins in root methanolic extracts was determined as described by Nakamura et al. (2003). For sample reactions, 100 μL root methanolic extract were mixed with 250 μL vanillin solution (1% in methanol) and 250 μL HCl 9 N. Control reactions were realized with 100 μL methanol, 250 μL vanillin solution (1% in methanol) and 250 μL HCl 9 N. Reactions were incubated 30 min at 30°C and the absorbance at 500 nm was determined. Absorbance of control reactions were subtracted to absorbance of sample reactions as described by Nakamura et al. (2003). Results were expressed as Absorbance units at 500 nm per g of fresh weight.

### Identification and Quantification of Flavonoids by LC-MS

Metabolites were extracted from hairy roots as described above (see section “Determination of Tannins With the Vanillin Method”). Identification and quantification of flavonoids was performed using an Agilent 1100 series High Performance Liquid Chromatography system coupled to Agilent 6510 accurate-mass Quadrupole-Time of Flight (Q-TOF) Mass spectrometer with ESI interface in negative ionization mode (Agilent Technologies, California, United States). A Zorbax SB-C18 column (3.1 × 150 mm, ϕ3.5 μm), equipped with a 2.1 × 12.5 mm ϕ 5 μm Zorbax Eclipse plus C18 precolumn (Agilent Technologies), was used at 35°C. The injected volume was 3 μL, the elution gradient was performed with binary solvent system composed of 0.1% formic acid in H_2_O (solvent A) and 0.1% formic acid in MeOH (solvent B) at a constant flow-rate of 0.35 mL.min^–1^. The gradient elution program was as follows: 0–3.0 min, 5% B; 3.0–23.0 min, up to 100% B; held for 10.0 min, followed by 7 min of stabilization at 5% B. LC-MS grade water, methanol and formic acid were purchased from Thermo Fisher Scientific (Illkirch, France).

The mass spectrometer operated by detection in scan mode with the following settings: drying gas 13.0 L.min^–1^ at 325°C; nebulizer pressure 35 psi; capillary voltage −3500 V, fragmentor 150 V. Negative mass calibration was performed with standard mix G1969-85000 (Agilent Technologies).

Data were acquired with Agilent MassHunter version B.02.00 software and processed with Agilent MassHunter Qualitative and Quantitative software version B.07.00. Absolute flavonoid contents were calculated from external calibration curves prepared with pure standards: catechin, epicatechin, and procyanidins B1 and B2 were purchased from Extrasynthese (Genay, France).

### Statistical Analysis

Data were analyzed by using a multifactorial ANOVA and a multiple comparison of means using the Tukey test (*p* ≤ 0.05) performed with R 3.3.2 software (R Development Core Team, 2016).

## Results

### Overexpression of *VvSWEET4* in *V. vinifera* cv. Syrah Hairy Roots Results in Enhanced Growth on Culture Media Supplemented With Sucrose or Glucose

In order to study the sugar transport function of VvSWEET4 in grapevine, the coding sequence of this transporter was placed under the control of the 35S CaMV promoter and used to create transformed hairy roots from stem tissue of *V. vinifera* cv. Syrah plantlets. The resulting hairy root lines (*VvSWEET4*_OX_) contained the Ri plasmid of *A. rhizogenes* strain A4 along with the binary vector containing the *35S::VvSWEET4* construct. Lines transformed with the Ri plasmid of *A. rhizogenes* strain A4 and the empty binary vector (pBin) as well as lines transformed only with the Ri plasmid (A4) were also generated as controls.

**FIGURE 1:**
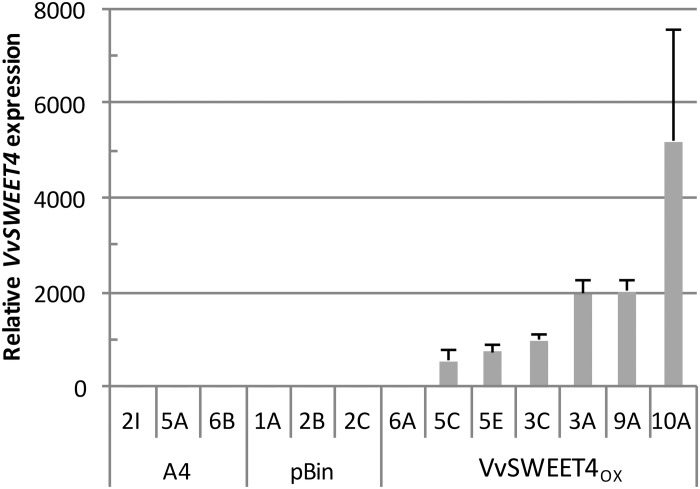
*VvSWEET4* expression levels in hairy root lines. Relative expression was measured 2 weeks after subculture in control lines transformed with the *A. rhizogenes* A4 strain alone (A4), the A4 strain with empty vector (pBin) and lines transformed with *35S::VvSWEET4* (*VvSWEET4*_OX_). Transcript levels of *VvSWEET4* were normalized to grapevine *GAPDH* and *EF1*α transcript levels. Relative expression indicates normalized expression levels in *VvSWEET4*_OX_ lines compared to normalized expression levels observed in control lines. Results are mean ± SD of three biological replicates.

**FIGURE 2:**
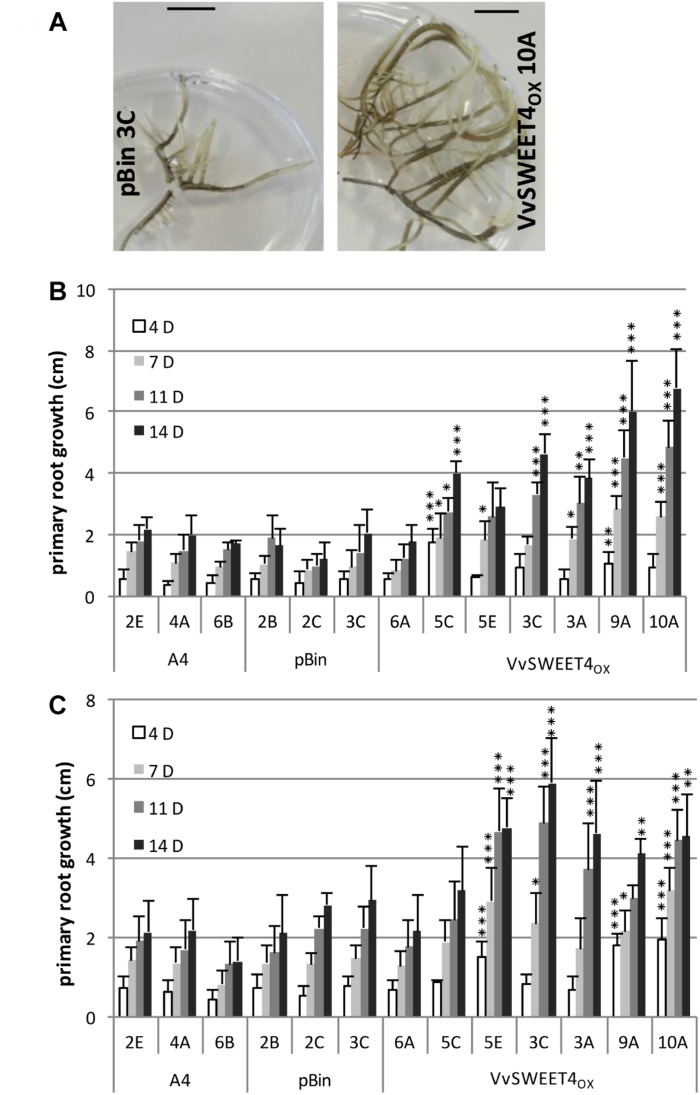
Growth of hairy root lines. **(A)** Representative pictures of pBin 3C and *VvSWEET4*_OX_ 10A lines after 3 weeks of culture on sucrose containing medium. Bar = 1 cm. **(B,C)** Growth of the hairy roots lines cultured on glucose **(B)** or sucrose **(C)** containing medium. Growth of the primary root (in cm) was measured at different times after subculture in control A4 and pBin lines and several *VvSWEET4*_OX_ lines. Mean ± SD was obtained from 6 biological replicates. Asterisks indicate a significant different mean root growth in *VvSWEET4*_OX_ lines compared to control A4 and pBin lines (ANOVA and multiple comparison of means, ^∗∗∗^*p* < 0.001, ^∗∗^*p* < 0.01, ^*^p < 0.1).

After obtention of transformed calli, several independent lines of hairy roots expressing different levels of the *VvSWEET4* transcript (*VvSWEET4*_OX_) were regenerated (Figure 1). During subcultures of the different hairy root lines, we noticed a faster growth of *VvSWEET4*_OX_ compared to controls (Figure 2A). Growth of the hairy root lines was studied in more detail by measuring the length increase of primary roots at different times after subculture on media supplemented with glucose (Figure 2B) or sucrose (Figure 2C). Roots from *VvSWEET4*_OX_ lines showed a greater length increase compared to roots from pBin and A4 lines, both on glucose and sucrose containing media, especially 11 and 14 days after subculture (Figures 2B,C). However, *VvSWEET4*_OX_6A, which had low levels of *VvSWEET4* transcripts did not show enhanced growth compared to control lines.

### *VvSWEET4*_OX_ Hairy Root Lines Have Higher Contents in Glucose and Fructose

**FIGURE 3:**
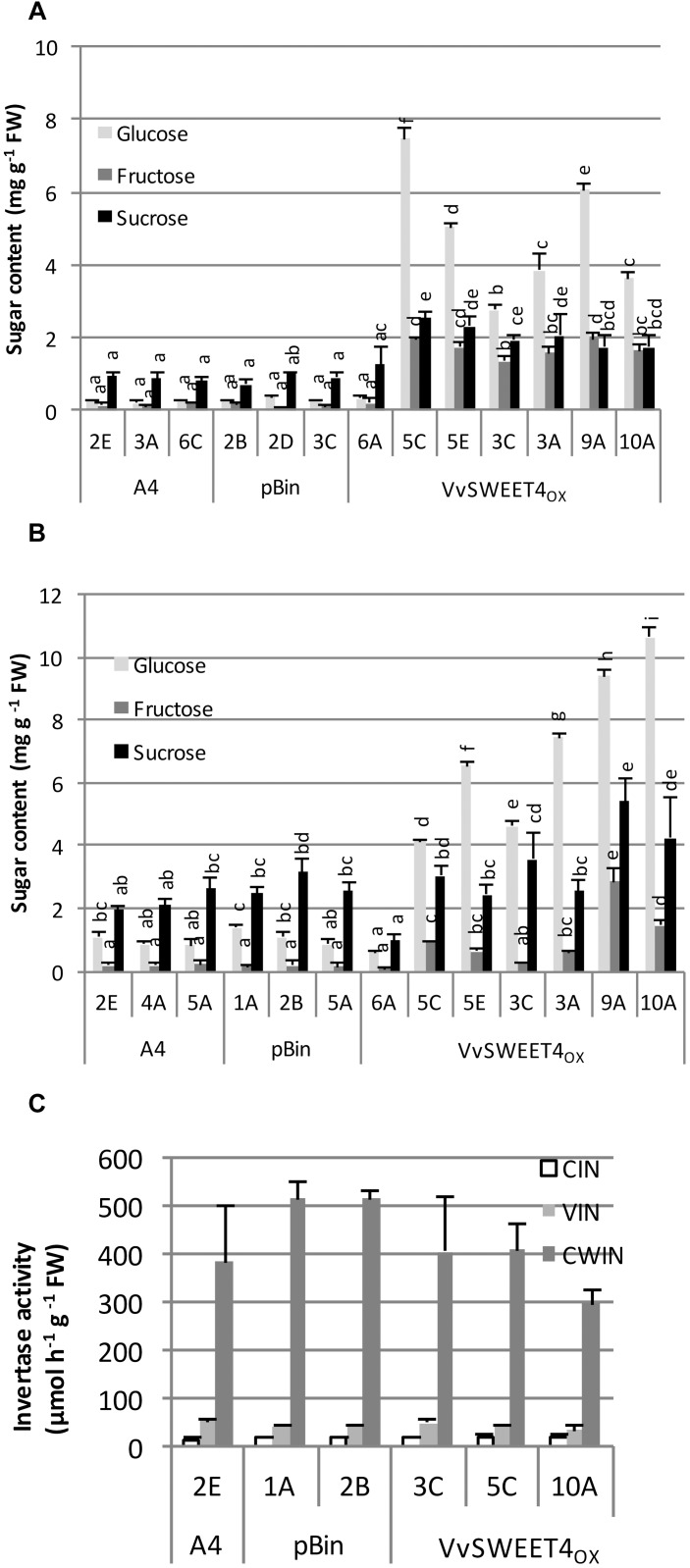
Sugar contents and invertase activities of hairy root lines. Levels of glucose, fructose and sucrose were measured in hairy root tips 2 weeks after subculture on sucrose **(A)** or glucose **(B)** containing medium. Data are the mean ± SD of 4 independent experiments. Means with different letters are significantly different at *p* ≤ 0.05 (Tukey Contrasts). **(C)** Cytoplasmic (CIN) vacuolar (VIN), and cell wall (CWIN) invertase activities. Data are the mean ± SD of three technical replicates. A second biological replicate with another set of lines produced similar results (Supplementary Figure S1). The different letters correspond to the different groups after the statistic Tukey Test (precized in the figure legend).

Increased growth rate observed in *VvSWEET4*_OX_ lines prompted us to determine their sugar contents 2 weeks after subculture. Levels of glucose, fructose and to a smaller extent sucrose were significantly higher in *VvSWEET4*_OX_ lines compared to pBin and A4 control lines when hairy roots were grown on a culture medium containing sucrose as carbon source (Figure 3A). Levels of glucose were especially high in *VvSWEET4*_OX_ lines, reaching up to 8 mg g^–1^ FW, except for *VvSWEET4*_OX_ 6A which had low levels of transgene expression and sugar levels comparable to those found in control lines. Sugar contents were also determined after 2 weeks of culture on a medium containing glucose as carbon source (Figure 3B). *VvSWEET4*_OX_ lines had significant higher contents in glucose except for *VvSWEET4*_OX_ 6A. Fructose levels also tended to increase but to a smaller extent, with the exception of lines *VvSWEET4*_OX_ 9A and 10A, where the increase was clear. When roots were grown on glucose as carbon source, sucrose contents were not significantly different in *VvSWEET4*_OX_ lines, except for *VvSWEET4*_OX_ 9A, and 10A (Figure 3B).

High levels of glucose and fructose found in *VvSWEET4*_OX_ hairy roots grown on sucrose containing medium are likely to result from extracellular invertase activity releasing glucose and fructose from sucrose, followed by hexose import through VvSWEET4 transport activity. Cytoplasmic, vacuolar and cell wall invertase activities were thus measured in hairy root lines. Although differences in activities could be observed between lines, no strong difference was found between *VvSWEET4*_OX_ and control lines (Figure 3C and Supplementary Figure S1).

### *VvSWEET4*_OX_ Hairy Root Lines Show Improved Glucose Absorption

Increased sugar levels in *VvSWEET4* overexpressors is likely to result from the sugar transport activity of VvSWEET4. We previously showed that VvSWEET4 is able to complement the growth on glucose medium of the yeast strain EBYVW4000, deficient in hexose transporters (Chong et al., 2014). Radiolabeled glucose absorption experiments were conducted on 2 control lines (A4 2B and pBin 2C) and 3 *VvSWEET4*_OX_ lines (5C, 9A, and 10A). Fragments of primary roots were incubated in medium containing high concentration of [^14^C]-glucose (10 mM) in order to create a steep gradient that favors glucose influx via facilitator transporters, such as SWEETs. After incubation, the amount of radiolabeled glucose was determined inside the roots.

**FIGURE 4:**
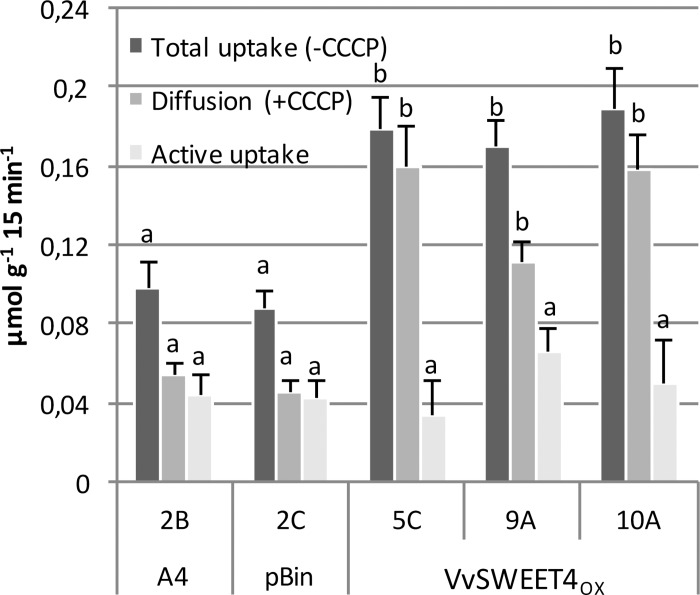
Radiolabeled glucose uptake in hairy roots. Five root fragments (30-day-old) from A4, pBin and *VvSWEET4*_OX_ lines were collected, and placed for 60 min (2 times) in equilibration buffer. Total glucose uptake of roots was measured after 15 min of incubation with 10 mM D-[^14^C]glucose. To measure glucose diffusion, CCCP (20 μM) was added into the equilibration buffer 10 min before addition of incubation buffer. Active glucose uptake results from the difference between total uptake and diffusion. Data are the mean ± SE of four independent replicates. Means with different letters are significantly different at *p* ≤ 0.05 (Tukey Contrasts).

Total glucose uptake rates were approximately two times higher in *VvSWEET4*_OX_ roots than in control roots (Figure 4). To discriminate between passive diffusion and proton-coupled transport, uptake assays were conducted in the presence of the protonophore carbonylcyanide m-chlorophenylhydrazine (CCCP), which causes an uncoupling of the proton gradient. Accordingly, proton-coupled transport (active uptake) is calculated as the difference between total glucose uptake and CCCP-independent uptake (diffusion). As shown in Figure 4, glucose retrieval into the control roots occurred by diffusion and active mechanisms with similar rates. In contrast, radiolabeled glucose retrieval by *VvSWEET4*_OX_ roots mainly occurred by a passive diffusion mechanism, since it was weakly inhibited by the protonophore CCCP. As expected, glucose uptake measured in the presence of CCCP was significantly increased in *VvSWEET4*_OX_ lines compared to controls. Moreover, active glucose uptake rates, resulting from the activity of hexose/H^+^ transporters was not significantly different between *VvSWEET4*_OX_ and control lines (Figure 4). Altogether, these results indicate that the expression of functional VvSWEET4 resulted in an increased glucose absorption into the roots through a facilitated diffusion mechanism.

### Overexpression of *VvSWEET4* in Hairy Roots Leads to Reduced Infection With *Pythium irregulare*, a Common Root Pathogen

**FIGURE 5:**
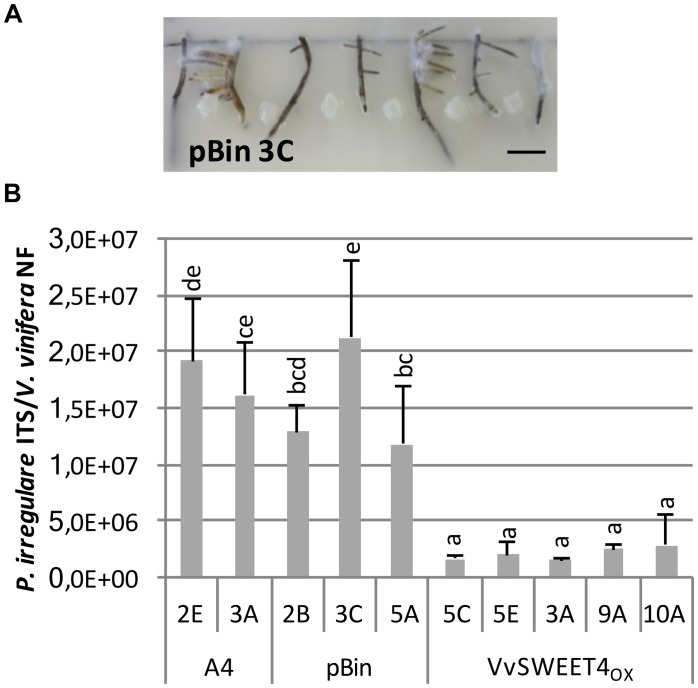
Overexpression of *VvSWEET4* in hairy roots reduces *Pythium irregulare* infection. Eight two-week-old root tips (2 cm length) from A4, pBin, and *VvSWEET4*_OX_ lines were placed 2 cm apart in square Petri dishes on water agar medium. Roots were inoculated by placing a 0.5 cm^2^ agar plug from the edge of a 1-week-old *P. irregulare* culture at equal distance between each root. Roots were harvested 3 days after inoculation. **(A)** Picture of hairy roots 3 days after inoculation. Bar = 1 cm. **(B)** qPCR was used to analyze the relative quantity of *P. irregulare* according to the abundance of fungal *ITS* sequence relative to grapevine *ACTIN* and *EF1*α genes. NF: normalization factor calculated from the geometric mean of grapevine *ACTIN* and *EF1*α relative DNA levels. Data represent means ± SD of four biological replicates each containing eight roots. Means with different letters are significantly different at *p* ≤ 0.05 (Tukey Contrasts).

We further investigated the consequences of improved glucose absorption and sugar levels in *VvSWEET4* overexpressors on the resistance to a common root pathogen, *P. irregulare*, which has been reported as a common pathogen in grapevine nurseries and is also one of the most prevalent *Pythium* species in vineyards in South Africa (Spies et al., 2011b). To favor infection of hairy roots by the pathogen, primary root fragments were placed on a water agar medium and inoculated with a *P. irregulare* mycelium plug placed between two root fragments (Figure 5A).

To monitor *P. irregulare* development in hairy roots, relative quantification of fungal and plant DNA in infected roots was realized by real-time quantitative PCR 3 days after inoculation as described in Berr et al. (2010). Relative quantities of *P. irregulare* DNA were markedly reduced in *VvSWEET4*_OX_ lines compared to A4 and pBin control lines (Figure 5B). These results show a less intense colonization of hairy roots with high *VvSWEET4* levels compared to A4 and pBin lines.

**FIGURE 6:**
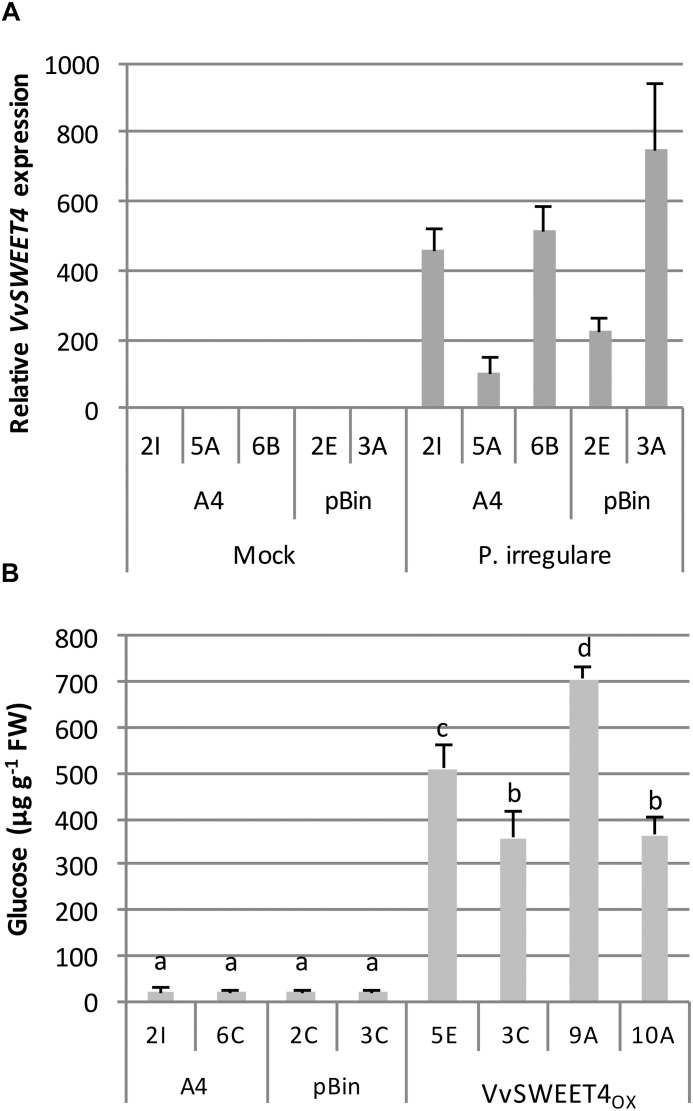
Endogenous *VvSWEET4* expression and glucose contents in hairy root lines after infection with *P. irregulare*. **(A)** *VvSWEET4* expression was measured in control hairy root lines (A4 and pBin) 3 days after infection with *P. irregulare*. Transcript levels of *VvSWEET4* were normalized to grapevine *GAPDH* and *EF1*α transcript levels. Relative expression indicates normalized expression levels in inoculated roots compared to normalized expression levels observed in mock-inoculated roots. Data represent means ± SD of three biological replicates. **(B)** Levels of soluble glucose were determined in hairy root lines 3 days after *P. irregulare* infection. Data represent means ± SD of 4 to 6 biological replicates. Means with different letters are significantly different at *p* ≤ 0.05 (Tukey Contrasts).

In order to better study the role of VvSWEET4 in the interaction with *P. irregulare*, we monitored the expression of *VvSWEET4* in control A4 and pBin lines 3 days after inoculation. As shown in Figure 6A, *VvSWEET4* was induced by *P. irregulare* infection suggesting that it could play a role in the interaction of grapevine roots with this pathogen. Levels of sugars were also measured in hairy roots 3 days after infection (Figure 6B). As a consequence of culture on water agar medium and pathogen infection, sugar levels were much lower compared to levels measured in hairy root lines on sugar containing medium (Figure 3). Levels of sucrose and fructose were especially low after infection and could not be accurately determined (data not shown). However, levels of glucose were significantly higher after infection in *VvSWEET4*_OX_ lines compared to controls (Figure 6B).

### Overexpression of *VvSWEET4* Is Associated With Constitutive Induction of Genes Involved in Flavonoid Biosynthesis and Higher Flavanol Contents

**FIGURE 7:**
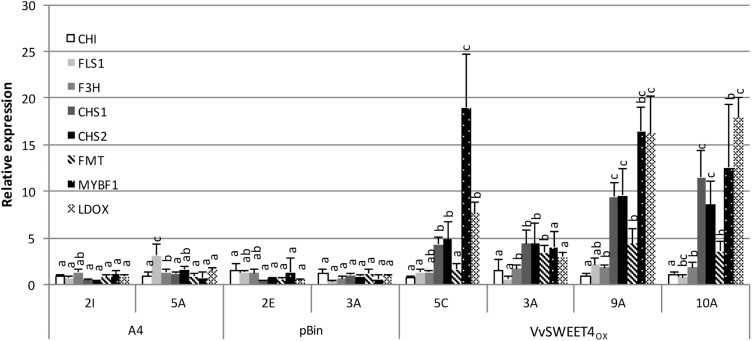
Expression analysis of grapevine flavonoid biosynthesis genes in hairy root lines. Expression of chalcone synthases (*VvCHS1*, *VvCHS2*), chalcone isomerase (*VvCHI*), flavanone-3-hydroxylase (*VvF3H*), flavonol synthase (*VvFLS1*), leucoanthocyanidin dioxygenase (*VvLDOX*), flavonoid-*O*-methyltransferase (*VvFMT*), and a R2R3-MYB transcription factor (*VvMYBF1*) was studied by qPCR in 2-week-old root tips from A4, pBin and *VvSWEET4*_OX_ lines. Transcript levels of the different genes were normalized to *Vitis vinifera GAPDH* and *EF1*α transcript levels. Relative expression indicates normalized expression levels in the different hairy root lines compared to the mean of the normalized expression levels measured in control A4 and pBin lines. Data are mean ± SD of three biological replicates. Means with different letters are significantly different at *p* ≤ 0.05 (Tukey Contrasts).

To know if improved resistance of *VvSWEET4*_OX_ lines could result from differences in defense gene activation, the expression of several defense genes was studied in healthy and infected roots. Expression of genes involved in the phenylpropanoid pathway (*VvPAL*, *VvSTS*), and genes encoding PR10 protein, callose synthase (*VvCalS*) or EDS1 signaling protein was similar in *VvSWEET4*_OX_ and control lines, both in healthy and diseased roots (Supplementary Figure S2). However, a significant higher constitutive expression of several genes involved in flavonoid biosynthesis (*VvCHS1*, *VvCHS2*, *VvLDOX*, and *VvFMT*) and of a MYB transcription factor (*VvMYBF1*) that regulates several genes involved in flavonoid synthesis in grapevine (Czemmel et al., 2009), was detected in most healthy hairy roots with high *VvSWEET4* levels (Figure 7).

**FIGURE 8:**
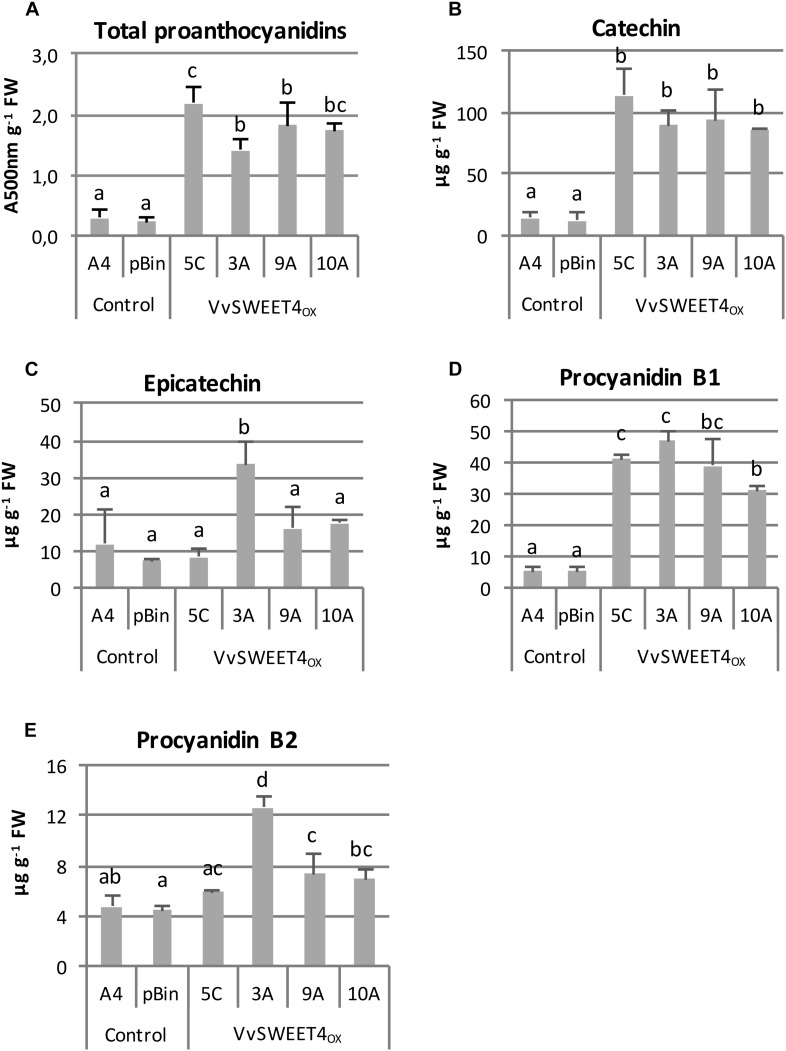
Flavanol contents in hairy root lines. **(A)** Total proanthocyanidin contents were determined in 3-week-old hairy roots by the vanillin method. Results are expressed as absorbance units at 500 nm per g of fresh weight. Data are mean ± SD of n biological replicates (*n* = 6 for controls, *n* = 4 for *VvSWEET4*_OX_). Means with different letters are significantly different at *p* ≤ 0.05 (Tukey Contrasts). **(B–E)**: quantification by LC/MS of catechin **(B)**, epicatechin **(C)**, procyanidin B1 **(D)** and procyanidin B2 **(E)** in 3-week-old hairy root lines. Data are mean ± SD of n biological replicates (*n* = 3 for controls, *n* = 4 for *VvSWEET4*_OX_). Means with different letters are significantly different at *p* ≤ 0.05 (Tukey Contrasts).

Higher expression of genes involved in flavonoid biosynthesis and regulation prompted us to investigate the flavonoid contents of *VvSWEET4* overexpressors. It has been previously reported that the main flavonoids in grapevine and especially hairy roots are proanthocyanidins (Huang et al., 2014). Proanthocyanidin contents were thus first determined using the colorimetric vanillin assay. As show in Figure 8A, total flavanol contents were significantly higher in *VvSWEET4*_OX_ hairy root lines compared to controls. Flavanols (catechin, epicatechin, procyanidins B1 and B2) were further quantified by LC-MS in hairy root extracts (Figures 8B–E). As shown on Figures 8B,D, contents in catechin and procyanidin B1 were significantly increased in *VvSWEET4*_OX_ hairy root lines compared to controls. Levels in epicatechin and procyanidin B2 were only significantly enhanced in *VvSWEET4*_OX_ 3A and were not significantly different in the other overexpressor lines compared to controls (Figures 8C,E).

## Discussion

### *VvSWEET4* Is an Hexose Passive Transporter

In this study, we demonstrated that *VvSWEET4* overexpression led to enhanced root elongation and higher contents in hexoses such as glucose and fructose. Similarly, overexpression of *AtSWEET4* in Arabidopsis also triggered an increase in plant size and in glucose and fructose contents (Liu et al., 2016). Enhanced root elongation likely results from higher sugar contents in roots. Indeed, several studies reported that root elongation depends directly on the carbon supply (Hennion et al., 2019).

SWEET transporters have been described as sugar uniporters, transporting sugars along the concentration gradient (Chen et al., 2010; Chandran, 2015). In our hairy root system, sugar uptake assays with radiolabeled glucose showed that VvSWEET4 functions in passive transport of hexoses (especially glucose) independent of the proton-motive force. In the hairy root system, high extracellular sugar levels favors hexose import mediated by VvSWEET4 in the roots resulting in enhanced contents in glucose and fructose. These results are consistent with the restoration of growth on glucose medium of the yeast EBYVW4000 mutant deficient in hexose transporters (Wieczorke et al., 1999) by *VvSWEET4* expression (Chong et al., 2014). Hairy roots overexpressing *VvSWEET4* cultured on sucrose-containing medium are characterized by significant higher glucose and fructose levels than controls. VvSWEET4 belongs to the clade II of SWEET transporter family, which has been described as monosaccharide (glucose, galactose, and fructose) but not sucrose transporters (Chen et al., 2010; Chandran, 2015). The presence of hexoses in hairy roots grown on sucrose medium is likely a consequence from extracellular cell wall invertase activity and a higher invertase activity in *VvSWEET4*_OX_ lines could explain their higher hexose contents. However, activities of cell wall, cytoplasmic and vacuolar invertases were not different between control and *VvSWEET4*_OX_ lines. It is probable that basal cell wall invertase activities found in control and *VvSWEET4*_OX_ hairy roots is sufficient to release glucose and fructose from sucrose. Enhanced hexose levels in *VvSWEET4*_OX_ lines thus results from enhanced import activity and not from enhanced sucrose cleavage activity.

### Enhanced Resistance of *VvSWEET4*-Overexpressing Hairy Roots to *Pythium* Infection

One of the disadvantages of the hairy root system is that it does not allow the regeneration of whole transformed plants, which represents a drawback for the test of pathogen resistance because it is limited to root pathogens. In our study, we thus chose to test the resistance of *VvSWEET4*_OX_ lines to a soil-borne root pathogen, *P. irregulare*, which exhibits a high virulence and represents one of the most important *Pythium* species in agriculture (Spies et al., 2011a). This necrotrophic pathogen is an important and widespread pathogen of grapevines in South Africa and could be a major cause of vine decline in nurseries and vineyards (Spies et al., 2011a,b). Interestingly, hairy roots overexpressing *VvSWEET4* were clearly less colonized after infection with *P. irregulare* compared to control lines. One hypothesis to explain this enhanced resistance is the high sugar content of these roots. It is known that plants with high sugar contents in their tissues are more resistant to several fungal diseases, a phenomenon called “high sugar resistance” (Morkunas and Ratajczak, 2014). Indeed, high sugar contents provide more energy for plant cells to fuel plant defenses, which are cost intensive. In accordance with high sugar levels in healthy *VvSWEET4*_OX_ hairy roots, glucose levels were also significantly higher compared to controls 3 days after infection with *P. irregulare*.

A recent study also reported the involvement of a SWEET transporter in the interaction between Arabidopsis and *P. irregulare* (Chen et al., 2015). The sugar facilitator AtSWEET2 is localized to the tonoplasts of cortex and epidermis root cells, and is involved in vacuolar glucose sequestration, thereby limiting the efflux of carbon from roots. The expression of *AtSWEET*2 in roots is induced by infection with the soil-borne pathogen *P. irregulare* and the loss of function *sweet2* mutant displays an enhanced susceptibility to this pathogen. This study revealed an important function for AtSWEET2 in modulating sugar secretion in the rhizosphere, that could support the growth of pathogenic root microorganisms (Chen et al., 2015). In the case of interaction with pathogenic bacteria, SWEET transporters have been mainly described as sugar effluxers favoring pathogen development (Chen et al., 2010; Chandran, 2015). In this study, we found that *VvSWEET4* expression is low in non-inoculated control hairy roots and is induced after *P. irregulare* infection. In Arabidopsis, promoter-GUS fusion analysis also showed that *AtSWEET4*, the *VvSWEET4* ortholog, is expressed in the stele of roots (Liu et al., 2016). It is thus possible that *VvSWEET4* plays a role in the interaction of grapevine roots with *P. irregulare.* VvSWEET4 is a plasma membrane localized transporter that would allow sugar leakage from plant cells into the apoplasmic space, which is favorable to pathogen development. Indeed, knockout mutants in *AtSWEET4* (the Arabidopsis *VvSWEET4* ortholog) were shown to be more resistant to *B. cinerea* infection (Chong et al., 2014). In the context of natural interaction of grapevine roots with *P. irregulare*, the induction of *VvSWEET4* expression following infection may facilitate pathogen development. Unexpectedly, hairy roots overexpressing *VvSWEET4* were found more resistant to *P. irregulare* but this resistance most likely results from high preexisting sugar levels rather than from impaired pathogen nutrition. Measurement of apoplastic sugar contents would be an interesting way to explain the various phenotypes after infection (Bezrutczyk et al., 2018).

### Overexpression of *VvSWEET4* and Consequences on Flavonoid Biosynthesis

In addition to their role in energy supply, several studies revealed that sugars can act as signaling molecules to trigger the expression of defense genes (Lemoine et al., 2013; Trouvelot et al., 2014). For example, it has been reported that sucrose induced the expression of *PR* genes (Thibaud et al., 2004; Gómez-Ariza et al., 2007). Morkunas and Bednarski (2008) also studied defense responses induced in *in vitro* cultured embryo axes of yellow lupine after infection by the hemibiotrophic fungus *Fusarium oxysporum*. They showed that several defense responses such as ROS production, peroxidase activities and lignin contents are more intense when embryos are nourished with exogenously supplied sucrose (Morkunas and Gmerek, 2007; Morkunas and Bednarski, 2008). In a recent study, Gebauer et al. (2017) showed a positive influence of sugar metabolism on the salicylic acid (SA) signaling pathway. They studied the *atsweet11/atsweet12* double knockout mutant, which presents constitutively elevated levels of soluble sugars, especially hexoses. Elevated sugar levels were associated with enhanced SA levels, priming of defense and signaling genes of the SA pathway and a better resistance to the hemibiotrophic fungus *Colletotrichum higginsianum* (Gebauer et al., 2017). However, in our study, no higher activation of defense genes regulated by SA in grapevine [*VvEDS1*, *VvPR1*, *VvPAL* (Chong et al., 2008)] was observed in *VvSWEET4*_OX_ lines compared to controls both in healthy and *P. irregulare* infected roots.

An important result from this study is that the flavonoid biosynthesis is enhanced in *VvSWEET4* overexpressors. Expression of several genes involved in the flavonoid pathway is constitutively enhanced in most *VvSWEET4*_OX_ hairy roots, whereas expression of other defense genes is not affected. We found that expression of chalcone synthases (*VvCHS1, VvCHS2*), putative flavonoid *O*-methyltransferase (*VvFMT*), leucoanthocyanidin dioxygenase (*VvLDOX*) and *VvMYBF1* transcription factor are constitutively up-regulated in most *VvSWEET4*_OX_ hairy root lines before infection, whereas expression of other flavonoid biosynthesis genes (Chalcone isomerase *VvCHI*, Flavanone-3-hydroxylase *VvF3H*, Flavonol synthase *VvFLS1*) is not affected. The flavonoid biosynthetic pathway genes are predominantly regulated at the level of transcription. The R2R3-MYB transcription factor VvMYBF1 is a specific transcriptional regulator of several genes involved in flavonoid synthesis in grapevine. VvMYBF1 was shown to activate promoters of genes involved in the general flavonoid pathway such as chalcone synthase and chalcone isomerase and also the *LDOX* promoter (Czemmel et al., 2009). Activation of *VvCHS*, *VvFMT* and *VvLDOX* in *VvSWEET4*_OX_ hairy root lines is thus consistent with the activation of VvMYBF1, which probably results from high sugar contents. However, the expression of *VvCHI* and *VvFLS1*, which are known targets of VvMYBF1 (Czemmel et al., 2009) was not significantly enhanced in *VvSWEET4*_OX_ hairy root lines. It is possible that in the hairy roots, activation of several MYB transcription factors by sugars does not lead to the same results as transient expression of a single MYB transcription factor in suspension cells of Chardonnay. In future studies, it will be also interesting to test the expression of other grapevine MYB transcription factors such as *VvMYBPA1* and *VvMYBPA2* reported to more specifically regulate the proanthocyanidin pathway (Bogs et al., 2007; Terrier et al., 2009). In several plant species, it has been demonstrated that sugars regulate the accumulation of flavonoids. Anthocyanins are a widespread class of plant flavonoids and their accumulation is modulated by sucrose, a well-characterized endogenous developmental signal (Solfanelli et al., 2006; Meng et al., 2018). Whole-genome transcript profiling reveals that sucrose treatment up-regulated the flavonoid and anthocyanin biosynthetic pathway and affects both flavonoid and anthocyanin contents (Solfanelli et al., 2006). In yellow lupine embryo axes infected with *F. oxysporum*, enhanced expression of flavonoid biosynthesis genes and higher flavonoid content were also reported when explants were supplied with sucrose (Morkunas et al., 2011). In Arabidopsis, the expression of a MYB transcription factor, AtMYB56, a potent regulator of anthocyanin accumulation, is induced by sucrose (Jeong et al., 2018). In addition, the expression of several structural genes and transcription factors involved in the anthocyanin biosynthesis pathway is regulated by sugars in petunia (Neta-Sharir et al., 2000).

In grapevine, it is known that genes involved in the phenylpropanoid pathway and anthocyanin biosynthesis are regulated by sugars (Lecourieux et al., 2014). First, there is a correlation between anthocyanin content of the grape berry and sugar accumulation at the post-veraison stages (Lecourieux et al., 2014). Second, the addition of sucrose, glucose or fructose to suspension cultures of *V. vinifera* cv. Gamay Fréaux increases the anthocyanin production up to 12-fold (Larronde et al., 1998). Similarly, increasing sucrose concentrations promote cell growth and phenylpropanoid biosynthesis in grape cell cultures obtained from *V. vinifera* cv. Barbera immature berries, leading to anthocyanin, catechin and stilbene accumulation or secretion in the culture medium (Ferri et al., 2011). Stimulation of the transcription of phenylpropanoid biosynthetic enzymes, such as phenylalanine ammonia lyase, chalcone synthase, chalcone-flavanone isomerase and stilbene synthase paralleled enhanced polyphenol production after sucrose treatment (Ferri et al., 2011). Other studies reported induction of flavonoid biosynthesis gene expression by exogenous sugar supply in grapevine. The expression of both *LDOX* (leucoanthocyanidin dioxygenase) and *DFR* (dihydroflavonol-4-reductase) was up-regulated by treatment of cv. Gamay Red cell cultures with sucrose (Gollop et al., 2001). The transcripts and protein amounts of F3H (flavanone 3-hydroxylase) protein also increased in grape berries incubated with different concentrations of glucose, fructose or sucrose (Zheng et al., 2009). In grapevine, the main flavonoids are proanthocyanidins which are major determinants for fruit and wine quality (Huang et al., 2014). We further showed that up-regulation of flavonoid biosynthesis genes results in higher total proanthocyanidin levels and especially enhanced contents in catechin and procyanidin B1 in *VvSWEET4*_OX_ lines compared to controls. Catechin and procyanidin B1 levels were comparable between *VvSWEET4*_OX_ 3A, 5C, 9A, and 10A. However, expression of flavonoid biosynthesis genes were lower in *VvSWEET4*_OX_ 3A and to a lesser extent 5C compared to expression in *VvSWEET4*_OX_ 9A and 10A. It is possible that expression of flavonoid biosynthesis genes observed in *VvSWEET4*_OX_ 3A is sufficient to result in higher flavanol contents. On another side, absence of correlation between flavonoid biosynthesis gene expression and flavanol contents could also be explained by the fact that gene expression was measured 2 weeks after hairy root subculture and flavanol contents was determined 3 weeks after subculture.

Overall, our results show that decreased susceptibility of *VvSWEET4*_OX_ lines to *P. irregulare* may be not caused by impaired sugar provision to the pathogen but could be a consequence of sugar induction of the flavonoid pathway. Flavonoids are secondary metabolites with antimicrobial and especially antifungal properties (Treutter, 2006). Study of barley mutants impaired in proanthocyanidin accumulation in the seed testa layer revealed that these compounds are necessary to resistance to *Fusarium* infection (Skadhauge et al., 1997). In Arabidopsis, plants with depleted flavonoid contents by silencing MYB transcription factors involved in flavonoid biosynthesis activation were more susceptible to infection with necrotrophic or hemibiotrophic pathogens. Conversely, stronger accumulation of flavonoids such as naringenin and kaempferol resulted in resistance to fungal pathogens (Camargo-Ramirez et al., 2018). In grapevine, study of calli with differential susceptibility to *P. viticola* revealed that resistant callus contained greater contents of gallocatechin derivatives (Dai et al., 1995). In addition, treatment of grapevine (cv Merlot) with benzothiadiazole (BTH) induced resistance to the gray mold *B. cinerea* and resistance was correlated with enhanced polyphenol contents in berry skins, especially the procyanidin fraction (Iriti et al., 2005).

## Conclusion

In conclusion, our study characterized the sugar transport activity of a grapevine SWEET transporter in grapevine hairy roots, confirming the usefulness of this system for the functional characterization of grapevine sugar transporters. Grapevine hairy roots circumvent the time-consuming process of generating stable transgenic lines in grapevine. *A. rhizogenes* transformation is indeed an interesting efficient alternative to embryogenic calli transformation with *Agrobacterium tumefaciens* followed by the regeneration of whole transformed plants, which has a particularly low efficiency in grapevine. Hairy roots have been already used as an efficient system for characterization of regulators of anthocyanin accumulation and transport in grapevine (Gomez et al., 2011; Matus et al., 2017). Using this homologous system, we showed that the VvSWEET4 transporter is an important component for sugar accumulation in grapevine cells and that sugar fluxes are crucial for pathogen resistance. Moreover, our study points out a key role of sugars as signaling molecules for the regulation of flavonoid biosynthesis. In plant-bioagressor interactions, modulation of sugar pools acting either as a source of energy or as regulators of defense responses such as plant secondary metabolite synthesis has critical consequences for pathogen resistance. Future work will try to elucidate the role of VvSWEET4 in the interaction with *P. irregulare* in natural conditions.

## Data Availability

All datasets generated for this study are included in the manuscript and/or the Supplementary Files.

## Author Contributions

JC, PM, EM, and SL conceived and designed the experiments. EM, HL, SL, JC, and M-LG carried out the experiments. EM, JC, PM, SL, and M-LG analyzed the experiments. M-LG contributed to the materials and analysis tools. JC, PM, and SL wrote the manuscript. JC acquired the funding.

## Conflict of Interest Statement

The authors declare that the research was conducted in the absence of any commercial or financial relationships that could be construed as a potential conflict of interest.
