# Supplementary material for: Overexpression of the VvSWEET4 Transporter in Grapevine Hairy Roots Increases Sugar Transport and Contents and Enhances Resistance to Pythium irregulare, a Soilborne Pathogen
Source: Front Plant Sci. 2019 Jul 9;10:884. doi: 10.3389/fpls.2019.00884 (PMC6629970; doi:10.3389/fpls.2019.00884)

## Slide 1
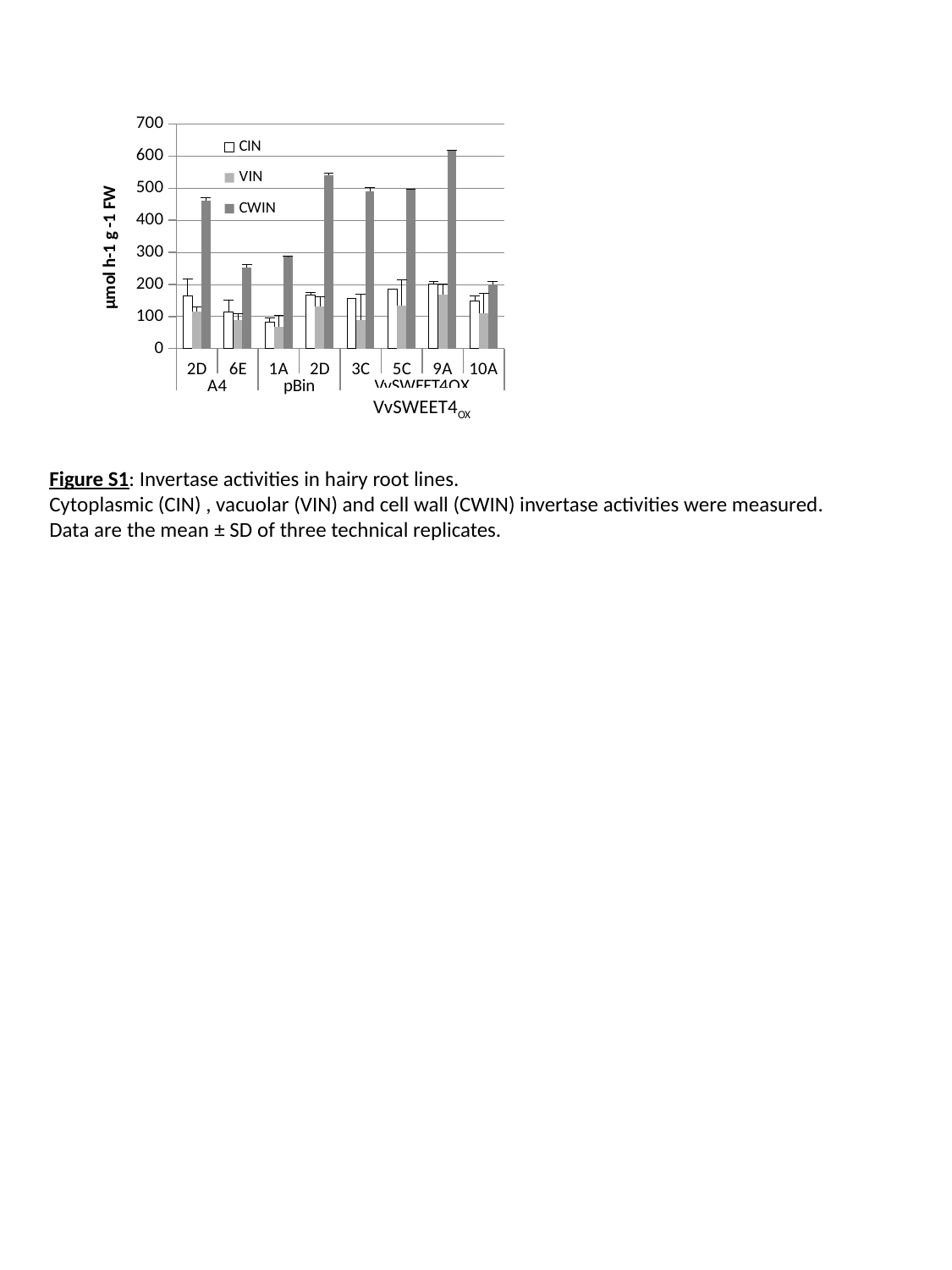

### Chart
| Category | CIN | VIN | CWIN |
|---|---|---|---|
| 2D | 164.79805087983974 | 114.14361201096892 | 461.537213783483 |
| 6E | 114.13163455170454 | 90.06906427828059 | 253.42962203790717 |
| 1A | 81.68028004667445 | 66.4948380069897 | 285.9144241869959 |
| 2D | 167.4997634740925 | 130.06296659465767 | 541.2238298747989 |
| 3C | 156.26169102728926 | 89.74586374005278 | 490.1393876607063 |
| 5C | 184.35188858329562 | 133.87458384442294 | 494.7033802235571 |
| 9A | 201.61217174878743 | 167.61941946634732 | 616.9488655882574 |
| 10A | 147.25824733375015 | 109.42646794285534 | 200.3266193194361 |VvSWEET4OX
Figure S1: Invertase activities in hairy root lines.
Cytoplasmic (CIN) , vacuolar (VIN) and cell wall (CWIN) invertase activities were measured. Data are the mean ± SD of three technical replicates.

## Slide 2
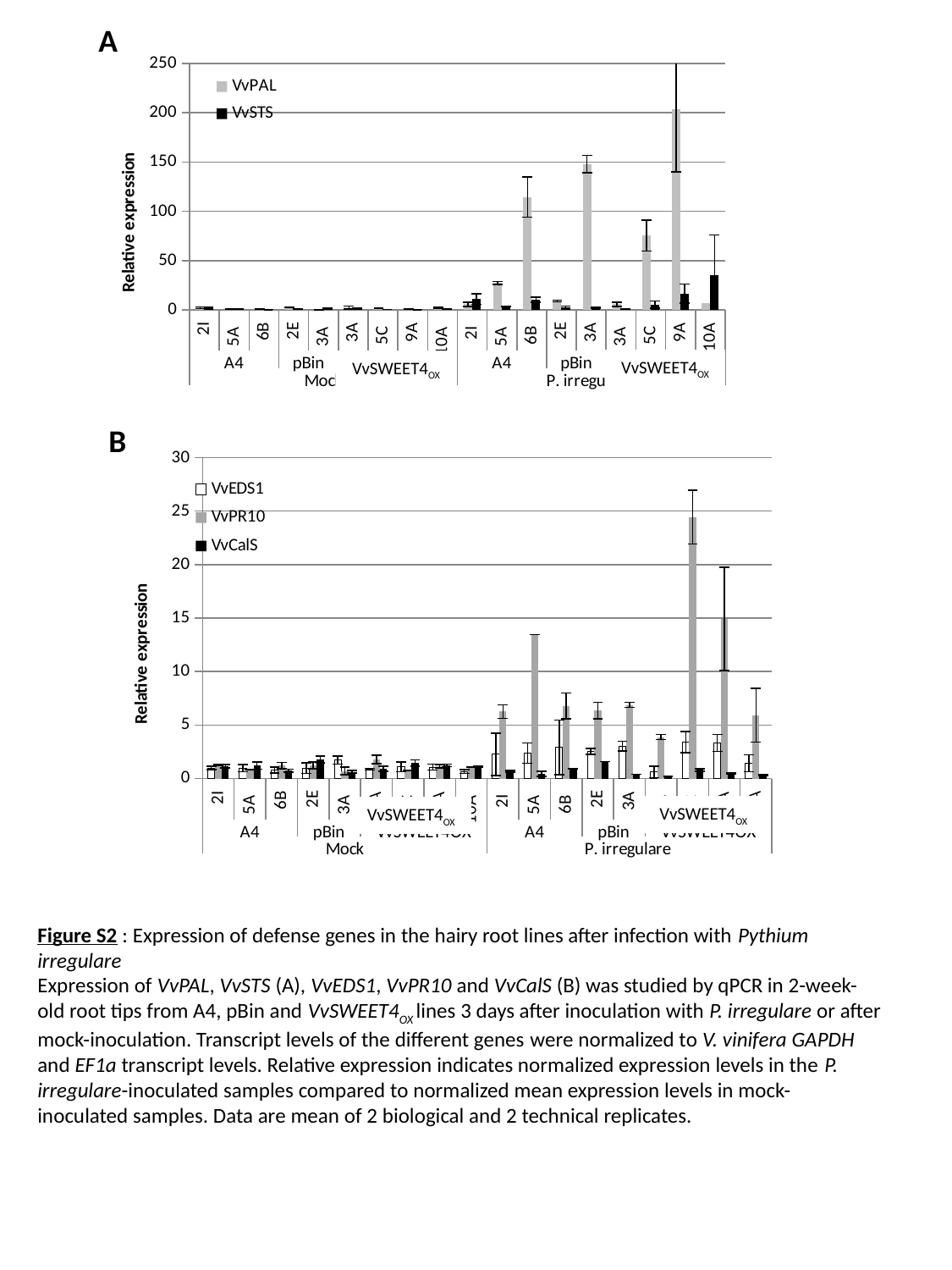

A
### Chart
| Category | VvPAL | VvSTS |
|---|---|---|
| 2I | 2.3549429917517632 | 2.5527503089344616 |
| 5A | 1.2763230358726958 | 0.6725059573848647 |
| 6B | 0.6489176544078045 | 0.49670067035925947 |
| 2E | 2.6030762831881753 | 0.9670671307690691 |
| 3A | 0.22449184440908296 | 1.502807291777722 |
| 3A | 2.6328138161783676 | 1.2596694731307776 |
| 5C | 2.0504697080313545 | 0.626889858835198 |
| 9A | 1.084493242760421 | 0.5507805977786165 |
| 10A | 2.3381243986424383 | 1.025269047840798 |
| 2I | 5.7862113206086905 | 11.049908366460494 |
| 5A | 27.29163612320454 | 3.3176672892208723 |
| 6B | 114.37819265732492 | 10.441114796542156 |
| 2E | 9.269483119065718 | 2.608951629272685 |
| 3A | 147.88655347283034 | 2.101914304325436 |
| 3A | 5.727171213315613 | 1.2509972257032018 |
| 5C | 75.34505360403101 | 5.681871885510341 |
| 9A | 203.93460969310783 | 16.76460084752427 |
| 10A | 7.280662226703469 | 35.51707834191083 |VvSWEET4OX
VvSWEET4OX
B
### Chart
| Category | VvEDS1 | VvPR10 | VvCalS |
|---|---|---|---|
| 2I | 0.9765826068829577 | 1.2615519298932103 | 1.1505367826829394 |
| 5A | 0.9755882124941664 | 0.8301327526883346 | 1.2020546465829594 |
| 6B | 0.7941317668909146 | 1.2121239084208744 | 0.7249610076111469 |
| 2E | 0.9728850891571127 | 1.2304789433794907 | 1.7927818790840782 |
| 3A | 1.733889588476315 | 0.7030633680579765 | 0.6253904593848318 |
| 3A | 0.8654855044416534 | 1.7922947996504848 | 0.9128309250674254 |
| 5C | 1.0910240758132055 | 0.7704197525932781 | 1.4491526284604013 |
| 9A | 1.0671844128936103 | 1.1460006069655977 | 1.2256121859996032 |
| 10A | 0.6404661020923751 | 1.0571465079610767 | 1.1245643835009793 |
| 2I | 2.2601929667443508 | 6.257057109904561 | 0.676417043081683 |
| 5A | 2.3830598594273913 | 13.477677455468667 | 0.44350865379121196 |
| 6B | 2.9085895185477715 | 6.790396332868708 | 0.9259809680063993 |
| 2E | 2.542103582830292 | 6.340405528176356 | 1.5711635149236898 |
| 3A | 3.0299845999321455 | 6.878486593457586 | 0.39806556424781514 |
| 3A | 0.6013685792308081 | 3.8788539222670626 | 0.20802186866223377 |
| 5C | 3.4075948771041964 | 24.44246031493804 | 0.8115680059541143 |
| 9A | 3.3415352236311455 | 14.930766343087033 | 0.497857727133435 |
| 10A | 1.4312904718238453 | 5.913736955890123 | 0.2953550103236484 |VvSWEET4OX
VvSWEET4OX
Figure S2 : Expression of defense genes in the hairy root lines after infection with Pythium irregulare
Expression of VvPAL, VvSTS (A), VvEDS1, VvPR10 and VvCalS (B) was studied by qPCR in 2-week-old root tips from A4, pBin and VvSWEET4OX lines 3 days after inoculation with P. irregulare or after mock-inoculation. Transcript levels of the different genes were normalized to V. vinifera GAPDH and EF1a transcript levels. Relative expression indicates normalized expression levels in the P. irregulare-inoculated samples compared to normalized mean expression levels in mock-inoculated samples. Data are mean of 2 biological and 2 technical replicates.

## Slide 3
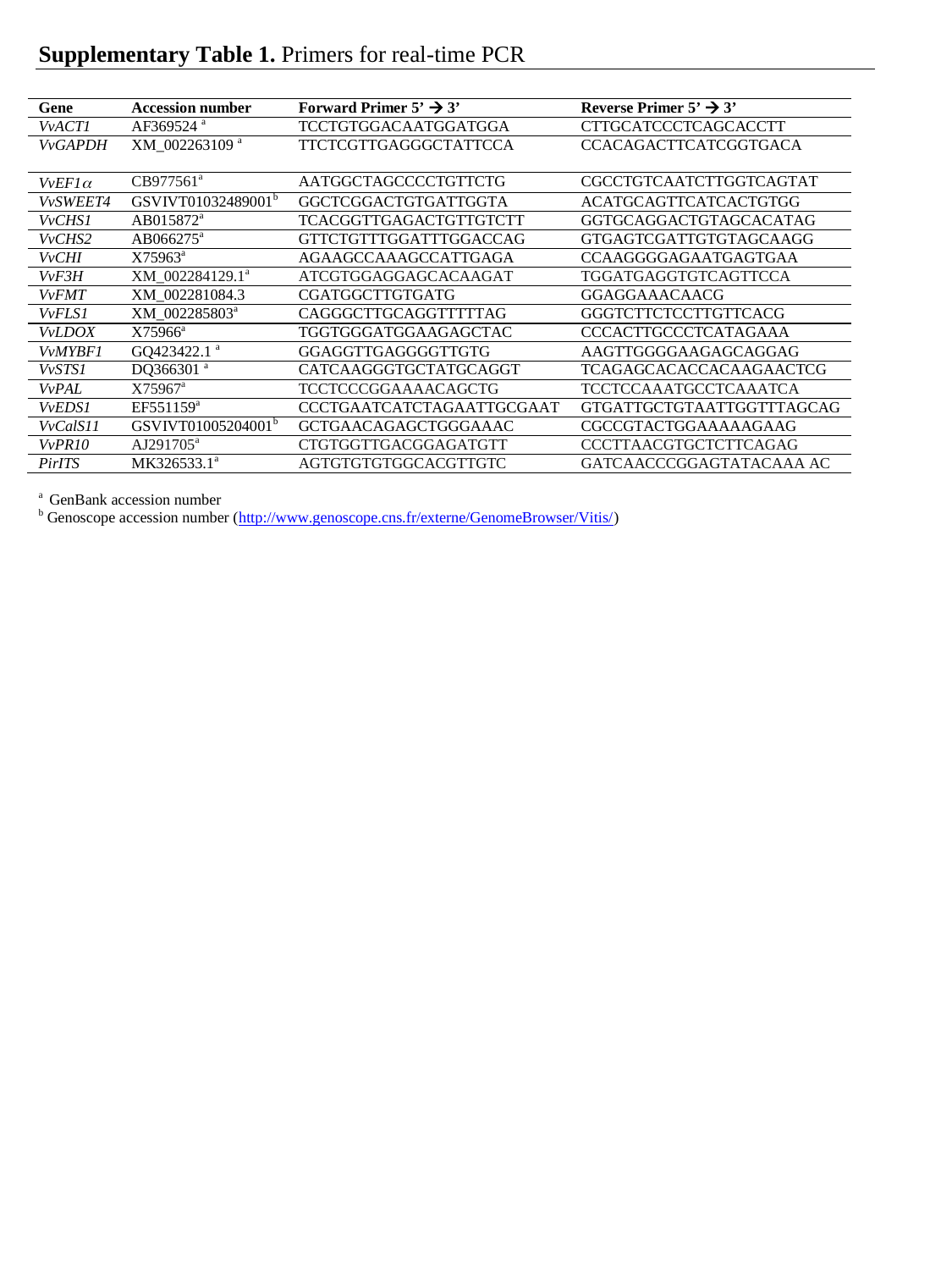

Supplement: Supplementary file 1 [file Presentation_1.PPTX]
